# Development and validation of a novel risk score for the detection of insignificant prostate cancer in unscreened patient cohorts

**DOI:** 10.1038/s41416-018-0316-2

**Published:** 2018-11-27

**Authors:** Lorenzo Dutto, Amar Ahmad, Katerina Urbanova, Christian Wagner, Andreas Schuette, Mustafa Addali, John D. Kelly, Ashwin Sridhar, Senthil Nathan, Timothy P. Briggs, Joern H. Witt, Gregory L. Shaw

**Affiliations:** 1 Prostatazentrum Nordwest, St. Antonius-Hospital, Klinik für Urologie, Kinderurologie und Urologische Onkologie, Gronau, Germany; 2 Department of Urology, Queen Elisabeth University Hospital, Glasgow, UK; 3 Wolfson Institute of Preventive Medicine, Barts and The London School of Medicine, Queen Mary University of London, Centre for Cancer Prevention, London, UK; 4 Department of Urology, University College London Hospital, London, UK

## Abstract

**Background:**

Active surveillance is recommended for insignificant prostate cancer (PCa). Tools exist to identify suitable candidates using clinical variables. We aimed to develop and validate a novel risk score (NRS) predicting which patients are harbouring insignificant PCa.

**Methods:**

We used prospectively collected data from 8040 consecutive unscreened patients who underwent radical prostatectomy between 2006 and 2016. Of these, data from 2799 patients with Gleason 3 + 3 on biopsy were used to develop a multivariate model predicting the presence of insignificant PC at radical prostatectomy (ERSPC updated definition^3^: Gleason 3 + 3 only, index tumour volume < 1.3 cm^3^ and total tumour volume < 2.5 cm^3^). This was used to develop a novel risk score (NRS) which was validated in an equivalent independent cohort (*n* = 441). We compared the accuracy of existing predictive tools and the NRS in these cohorts.

**Results:**

The NRS (incorporating PSA, prostate volume, age, clinical T Stage, percent and number of positive biopsy cores) outperformed pre-existing predictive tools in derivation and validation cohorts (AUC 0.755 and 0.76, respectively). Selection bias due to analysis of a surgical cohort is acknowledged.

**Conclusions:**

The advantage of the NRS is that it can be tailored to patient characteristics and may prove to be valuable tool in clinical decision-making.

## Introduction

Insignificant prostate cancer (PCa) can be defined as a cancer, which will not affect the patient during the natural course of his lifetime.^1^ Attempts have been made to define clinically insignificant PCa, based on pathological examination of radical prostatectomy specimens and analysis of recurrence rates of these tumours. Several definitions have been formulated. For example, Wolters et al.^2^ analysed the data from the European Randomised Study of screening for PCa (ERSPC)^3^ to define insignificant PCa as organ-confined Gleason 3 + 3 tumours, with no grade 4 or 5, the largest tumour having a volume ≤ 1.3 cm^3^ and a total tumour volume of ≤2.5 cm^3^. With the intent to reduce overtreatment of such patients, active surveillance (AS) has become established as a treatment option for selected patients thought to harbour insignificant PCa.^1^ In order to correctly identify possible candidates for AS, a number of predictive tools have been developed to predict low-risk PCa based on clinical parameters, such as clinical T-stage, PSA, PSA-density (PSAD), prostate volume, prostate biopsy (Gleason grade and percentage of positive cores (PPC)), and patient age.^4–13^

The aforementioned predictive tools have mostly been developed in PSA screened patient cohorts. It is known that PSA screening results in stage and grade migration with smaller low-grade tumours being detected.^14–16^ In Europe, PSA testing is not performed widely. In the United States PSA testing is more common than in Europe, but less common than it was prior to the USPSTF ruling against PSA screening.^17^ This raises the question of whether the existing predictive tools, which are currently used to select patients for AS programmes, are sufficiently accurate when they are used in unscreened patient cohorts. Diagnostic inaccuracy means that confirmatory biopsy is recommended for men embarking on AS.^18^

## Objectives

The primary aim of the study was to develop a purpose-specific novel risk score (NRS) for use in daily clinical practice that can identify insignificant PCa in unscreened patient cohorts.

Furthermore, the NRS was designed to give a risk score relating to a risk ratio of an individual patient harbouring a significant PCa, rather than the binary output of existing predictive tools, thereby aiding in the decision-making process on whether an individual may be suitable or unsuitable for AS or treatment. The secondary aim of the study was to test the accuracy of existing predictive tools in unscreened patient cohorts and to compare their performance against that of the NRS in an independent unscreened patient cohort.

## Population and methods

### Study population and data collection

**Fig. 1:**
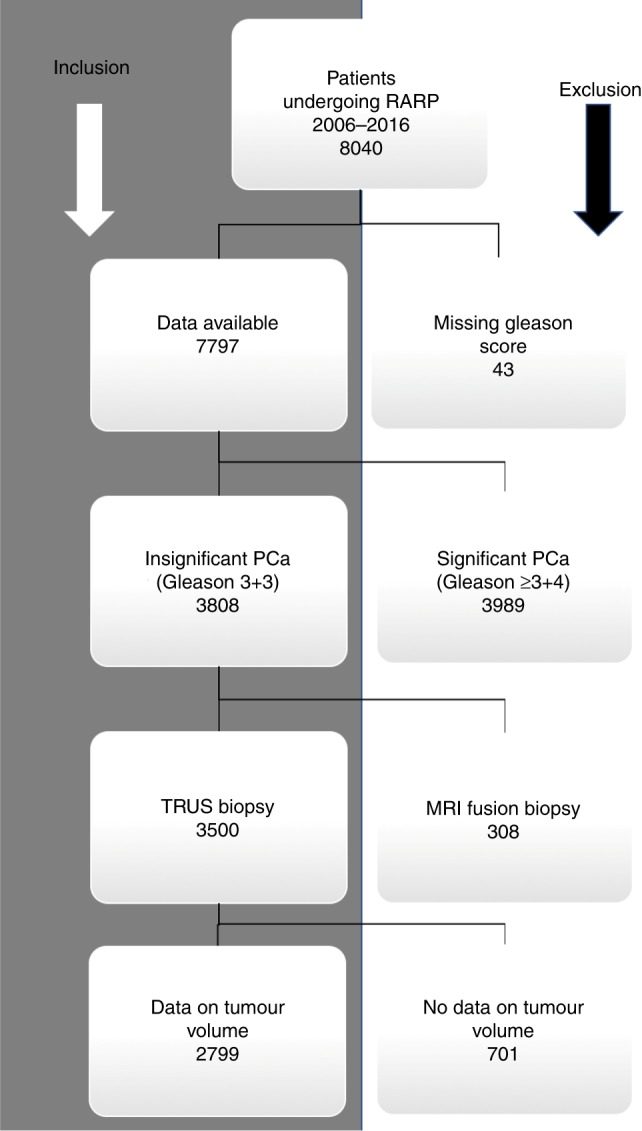
Flowchart for patient inclusion in data analysis

We analysed the data from 8040 consecutive unscreened patients who underwent radical robotic prostatectomy (RARP) at a German tertiary referral centre between February 2006 and January 2016. Data was prospectively collected after patients had given written informed consent for data collection. Full ethical approval was obtained from the University of Münster, Germany. Data on PSA, patient age, Gleason score on biopsy, PPC, prostate volume on trans-rectal ultrasound (TRUS), clinical T-stage and pathology findings after prostatectomy was available for 7797 patients. Amongst these we identified 3808 patients who had been diagnosed with Gleason 3 + 3 PCa. Of these, 308 patients that had been diagnosed via MRI-based fusion biopsy were excluded, as the different sampling method may act as a possible confounder. Further 701 patients were excluded for missing data on tumour volume on pathology. The remaining 2799 patients were included for final analysis (flowchart shown in Figure 1). These patients’ pathology findings after prostatectomy were stratified according to the updated ERSPC PCa risk criteria^2^ and were used to develop the NRS (derivation cohort). The validation cohort consisted of 430 unscreened men who underwent RARP for Gleason 3 + 3 PCa in two tertiary referral centres in the UK and for whom the same clinical data as the derivation cohort was available.

### Statistical methods

The statistical analysis was performed by means of the *R* software version 3.4.1.^19^ All tests were two sided and *p*-values < 0.05 were accepted as statistically significant. No *p*-value adjustment was performed for multiple comparisons.

#### Handling of missing data

There were four missing values for the number of positive biopsy cores that were imputed by the study populations’ median value of three. Similarly, there were four missing values for the PPC, which were imputed by the median of 22. The 24 missing DRE values were imputed as the median stage T1c.

All statistical analyses were performed on the imputed data set.^20^

#### Development of the NRS

Multivariate logistic regression with elastic net regularisation was used with the following preoperative clinical findings as predictors: log(1 + PSA), prostate volume on TRUS, age at diagnosis, DRE-Stage, PPC and number of positive cores (NPC). The outcome variable was insignificant PCa found in the prostatectomy specimens, with insignificant PCa = 1 and significant PCa = 0 according to the updated ERSPC definition.^3^

A 10-fold cross-validation was applied using the R-package glmnet. All variables were selected within minimum and one standard error binomial deviance. Spearman’s correlation coefficient between all six clinical predictors was computed (Table 1).

**Table1:** candidate clinical diagnostic factors in the derivation and validation datasets

Variable name	Variable	Definition/units	Median (IQR)	
			Derivation	Validation
Age	Age	year	63.93 (68.94–58.96)	64.00 (68.00– 59.00)
log(1 + baseline PSA)	PSA	ng/ml	2.07 (2.40–1.82)	2.08 (2.35–1.81)
TRUS prostate volume	TRUS	cc	40 (55.00–31.00)	43.5 (58.0–32.0)
DRE stage	Stage	cT1a = 4, cT1b = 5, cT1c = 6, cT2a = 7, cT2b = 8, cT2c = 9	6 (7.00–6.00)	6 (7.00–6.00)
Percentage of positive cores	PPC	%	22.00 (40.00–11.00)	30.8 (50.00–16.67)
Number of positive cores	NPC	Number	3.00 (4.00–1.00)	3 (5.00–2.00)

The NRS was developed as the weighted sum of the clinical variables in the multivariate model; where the weights are the shrunken regression coefficients via the penalised maximum likelihood of the elastic-net regularisation.

#### Determination of accuracy of the NRS and comparison with existing risk scores

The performance of the NRS was assessed by the area under the curve (AUC) with 95% confidence intervals (CI) using the Delong method.^21^ Sensitivity and specificity were estimated at selected cut-off values. To evaluate the applicability of the NRS at different PSA levels the performance test was repeated for PSA subgroups (groups were: PSA 0–6, 6–10, 10–20, and 20–100 ng/ml).

Sensitivity and specificity with 95% confidence interval (95% CI) were computed for a range of published predictive tools designed to predict insignificant PCa. The performance of the NRS was compared with the best performing existing risk scores in terms of specificity and sensitivity. This was done by calculating the AUC’s of our NRS and of the previously existing risk scores by applying them to the derivation and validation cohorts of our study.

#### External validation

Data from 430 unscreened patients with preoperative characteristics of insignificant disease (Gleason 3 + 3 on biopsy, clinically organ confined) were used for validation of the NRS. The NRS was computed in the validation dataset exactly as reported for the derivation dataset. A calibration slope was estimated to measure the amount of occurred overfitting in the development of the NRS.

The AUC for receiver operating characteristics (ROC) was computed as a discrimination index to assess the overall ability of the NRS to separate insignificant from significant PCa in both derivation and validation datasets. DeLong’s test was performed to compare the two ROC curves of the NRS in the derivation and validation datasets.

## Results

All six preoperative clinical variables were statistically significant in a multivariate logistic model with insignificant PCa = 1 and significant PCa = 0 (Table 2). The highest observed correlation was between PPC and NPC (Spearman *r* = 0.897, *p* < 2.2 × 10^−16^). Weak correlations were observed between all other predictors.

**Table 2:** Multivariable logistic regression

	OR (95% CI)	Wald *z*-value	*p*-value
log(1 + PSA)	0.282 (0.230, 0.346)	−12.128	<2.2 × 10^−16^
Prostate volume on TRUS	1.023 (1.018, 1.027)	10.409	<2.2 × 10^−16^
Age	0.949 (0.937, 0.961)	−8.028	8.88 × 10^−16^
Stage	0.733 (0.651, 0.826)	−5.101	3.38 × 10^−07^
PPC	0.984 (0.976, 0.992)	−3.920	8.85 × 10^−05^
NPC	0.922 (0.855, 0.995)	−2.088	0.037
LR *χ*² (d.f., *p*-value)	561.861 (6, <2.2 × 10^−16^)		

*N* = 2799, *N*-insignificant PCa = 1045. Odds ratios (OR) and 95% confidence interval (CI) are presented for one unit change

*PPC* percentage of positive cores, *NPC* number of positive cores

The NRS was developed as the weighted sum of the clinical variables in the multivariate model; where the weights are the shrunken regression coefficients via the penalised maximum likelihood of the elastic-net regularisation:

NRS = −1.227*log(1 + PSA) + 0.022*TRUS − 0.050*Age − 0.301*Stage − 0.016* PPC − 0.08*NPC.

Supplementary Figure 1 shows the distribution of the NRS in the derivation and validation datasets. Supplementary Table 1 shows the study populations’ distribution by PCa risk group and clinical stage on DRE .

**Table 3:** Concordance and discordance between prediction of the existing risk scores and pathology findings, with resulting sensitivity and specificity with 95%CI

Risk score	AUC	TN	FN	FP	TP	Specificity (95%CI)	Sensitivity (95%CI)
Carter^5^	0.611	1579	709	175	336	0.900 (0.885, 0.914)	0.322 (0.293 0.351)
Soloway^4^	0.627	1344	535	410	510	0.766 (0.746, 0.786)	0.488 (0.457, 0.519)
Eastham^6^	0.634	1134	397	620	648	0.647 (0.624, 0.669)	0.620 (0.590, 0.650)
Carroll^13^	0.626	1038	355	716	690	0.592 (0.568, 0.615)	0.660 (0.631, 0.689)
Babaian^7^	0.614	1026	374	728	671	0.585 (0.561, 0.608)	0.642 (0.612, 0.671)
Parker^8^	0.584	474	106	1280	939	0.270 (0.250, 0.292)	0.899 (0.879, 0.916)
Very low^a^	0.628	1316	515	438	530	0.750 (0.729, 0.770)	0.507 (0.476, 0.538)

*TN* true negative, *FN* false negative, *FP* false positive, *TP* true positive

^a^Corresponding to the common definition of very low PCa, often used to enrol patients in active surveillance protocols

Our NRS yields a range of cut-off values that can be selected from and which will produce different specificity and sensitivity levels. We therefore chose two cut-off values that we saw as most suited for use in clinical practice and which were used for comparison with the previously existing risk scores.

**Fig. 2:**
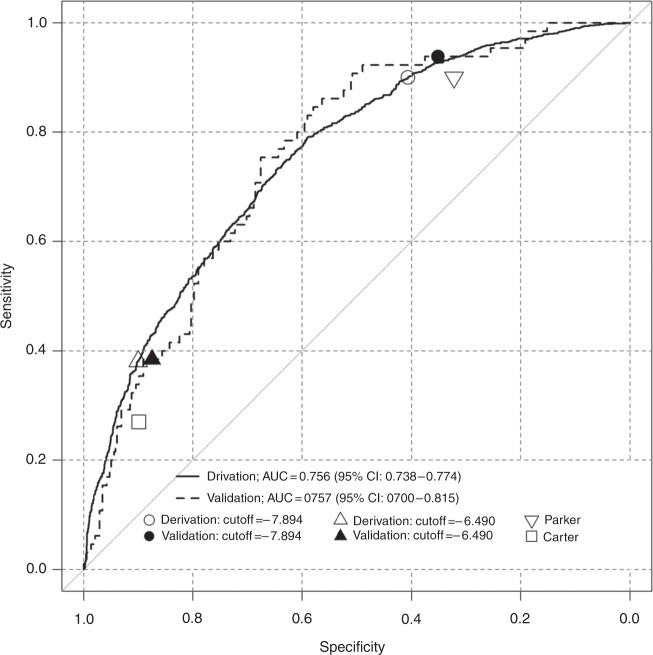
ROC curves of the novel predictive score in the derivation and validation datasets. A cut-off value of −8.013 gives sensitivity of 0.9 (95% CI: 0.881–0.918), with a specificity of 0.406 (95% CI: 0.383–0.430) on the derivation dataset (white circle). In the validation dataset this cut-off value gave a sensitivity of 0.938 (95%CI: 0.850–0.983) and a specificity of 0.351 (95% CI 0.303–0.402) (up-pointing white triangle). A cut-off value of −6.600 gives a specificity of 0.9 (95% CI: 0.885–0.914), with a sensitivity of 0.379 (95% CI: 0.349–0.409) on the derivation dataset (black circle). In the validation dataset this cut-off value gave a specificity of 0.875 (95%CI: 0.837–0.907) and a sensitivity of 0.385 (95% CI 0.267–0.514) (black triangle). The down-pointing white triangle shows the performance of the Parker score (most sensitive amongst previously existing risk scores) with a sensitivity of 0.899 (95% CI: 0.879–0.916) and a specificity of 0.270 (95% CI: 0.250–0.292). The white square shows the performance of the Carter score (most specific amongst previously existing risk scores) with a specificity of 0.900 (95% CI: 0.885–0.914) and a sensitivity of 0.322 (95% CI: 0.293–0.351)

In particular, we chose one cut-off value with high sensitivity (cut-off value: −8.013) and one with a high specificity (cut-off value: −6.600), as we thought that these thresholds would be best suited to reduce overtreatment, or to reduce the risk of missing a significant cancer, respectively (Fig. 2).

### External validation

The calibration slope, which is the regression coefficient on the NRS in the validation dataset, was 1.001 (*p*-value = 0.993), demonstrating no statistically significant overfitting, with good discrimination in the validation dataset.

### Determination of accuracy of the NRS

The ability of the NRS to separate insignificant PCa from significant PCa in the derivation and validation cohorts was measured through the AUC for ROC. The accepted rule of thumb is that an AUC below 0.7 indicates poor discriminative power; an AUC of 0.7–0.8 indicates acceptable discrimination; and above 0.8 indicates excellent discriminative ability.^21^

The NRS showed to be a good predictor in the derivation and validation datasets, with AUCs of 0.756 (95%CI: 0.738–0.774) and 0.758 (95%CI: 0.701–0.816), respectively (Fig. 2). The PSA subgroup analysis demonstrated that the NRS performed well across all PSA subgroups and may also be used in patients with a high PSA value (supplementary Figure 2).

DeLong’s test was used to compare the ROC curves of the NRS in the derivation and validation datasets. No statistically significant difference (*p*-value = 0.943) between the two AUC values was seen. The selected cut-off value of −8.013 gives a sensitivity of 0.9 (95% CI: 0.881–0.918), with a specificity of 0.406 (95% CI: 0.383–0.430) on the derivation dataset. In the validation dataset, this cut-off value has a sensitivity of 0.938 (95% CI: 0.850–0.983) and a specificity of 0.351 (95% CI 0.303–0.402). The cut-off value of −6.600 gives a specificity of 0.9 (95% CI: 0.885–0.914), with a sensitivity of 0.379 (95% CI: 0.349–0.409) on the derivation dataset. In the validation dataset, this cut-off value gives a specificity of 0.875 (95% CI: 0.837–0.907) and a sensitivity of 0.385 (95% CI 0.267–0.514) (Fig. 2).

### Comparative analysis of the NRS against existing risk scores

When we analysed the predictive power of our NRS and that of the existing risk scores by applying them to our study’s population, the NRS outperformed all previously existing risk scores.

This is demonstrated in Fig. 2, where the performance as measured by ROC curves-, sensitivity and specificity of the two selected thresholds of the NRS are compared against the best performing existing risk scores in terms of specificity^5^ and sensitivity^8^. None of the pre-existing risk scores reached the AUC threshold of 0.7 or higher to be classified as a good predictor. The inclusion criteria of the previously existing risk scores are summarised in supplementary Table 2. The the accuracy of pre-existing risk scores is summarized in Table 3.

## Discussion

Clinical decision making regarding the best course of action to take in patients who have what appears to be an insignificant form of PCa at diagnosis is a challenging task. The risk of overtreatment must be weighed against the risk of unnecessary biopsies and of possibly delaying necessary treatment. Data from the most mature AS cohorts demonstrate safety of an inclusive approach.^9, 22, 23^ However, 30% of patients on AS will require radical treatment within 5 years and, whilst an attempt at curative surgery might have been appropriate at the outset, it may no longer be appropriate as the patient ages. The degree to which diagnostic inaccuracy at the outset, or tumour evolution during the course of AS contribute to this failure of AS is an unknown, although the evolving data regarding inaccuracy of prostate biopsy suggest that undergrading and understaging at diagnosis pay a considerable part.^24^

The decision to start and continue on AS is complex and should be as well informed as possible. Existing predictive tools are of limited use as they have often been developed in patients that were screened for PCa, which is becoming increasingly less common. Our work shows that in unscreened patient cohorts our NRS outperforms the pre-existing clinical predictors. In addition, the existing tools by which patients are selected for AS give binary outcomes, meaning that patients are either deemed as low-risk patients, and therefore suitable for AS, or not. Our NRS allows the clinician to select an appropriate threshold that can best suit the patients’ needs. In practical terms the treating doctor can decide to choose a threshold of the NRS, which will either favour specificity or sensitivity. By choosing a threshold with high specificity, the clinician will minimise the risk of missing a significant PCa (for example, in a patient with long life expectancy or in whom anxiety means it is important to be sure that a more sinister cancer is not being overlooked). Conversely, choosing a threshold with higher sensitivity might be better suited to minimise the risk of unnecessary invasive treatment (as might be the case in a patient with lower life expectancy, who feels distressed about radical treatment options and who would prefer to undergo AS).

Given that our analysis showed poor predictive power of the existing risk scores when applied to our study’s unscreened patient cohorts, this should be considered in the process of clinical decision making; hence, a risk score that expresses a probability of risk, rather than a binary “yes/no” outcome, may be better suited to make informed decisions with the patients. Finally, with the exception of the risk score by Carter et al.^5^ that uses PSA density, all other existing risk scores are limited by their applicability within maximal PSA ranges (Supplementary Table 2), thereby excluding the possibility that a patient may hypothetically have insignificant PCa with a PSA > 15 ng/ml, regardless of what their prostate volume is.

PSA subgroup analysis showed that our NRS can be used without the limitation of maximum PSA levels (Supplementary Figures 2 and  3).

Our NRS should not be compared to well-known PCa risk scores and nomograms, such as the ones by Kattan et al.^25^ and D’Amico et al.^26^, which respectively, predict the likelihood of disease recurrence, or progression, or categorise patients into risk groups after radical treatment; nor should it be confused with other predictors such as the CAPRA score, which predicts an individual’s likelihood of metastasis, cancer-specific mortality, and overall mortality based on biopsy results and clinical parameters. The NRS we have developed is designed to focus more closely on those with low-risk disease and identify which of those with characteristics suggesting insignificant PCa will actually have a significant form of PCa. It may be that more accurate selection of patients for AS decreases the risk of misdiagnosis or progression, meaning that the need for repeated prostate biopsy during AS would be obviated and radical treatment, where necessary, would not be delayed.

We must however underline that our NRS is designed solely to identify insignificant PCa (according to the updated ERSPC definition). Whilst it outperforms current predictive tools, and can therefore be of aid to clinical decision making, the decision on whether a patient should undergo radical treatment or AS, is a decision to be made by the clinician and the patient after thorough counselling.

An online calculator where the odds ratio of a patient having a significant PCa is presented based on individual patient characteristics can be found here:

https://www.evidencio.com/models/show/1391

We excluded patients diagnosed via targeted-MRI-guided biopsies, as the different sampling methods may act as a possible confounder. We recognise the fact that the NRS does not currently include MRI findings, whilst mpMRI, fusion imaging and targeted biopsies are establishing themselves as valid tools in everyday clinical practice. However, it must be noted that MRI diagnostics for PCa are costly and are not yet consistently reimbursed in all healthcare settings. By including widely available diagnostic methods such as PSA, TRUS and DRE, our NRS can currently be used in most clinical setting throughout the world. Furthermore, to further increase its accuracy, we plan to extend the scope of our NRS to include MRI findings, fusion biopsy findings and targeted biopsies in the near future.

We acknowledge the inherent selection bias due to analysis of surgical cohorts, however this remains the only setting in which the actual tumour burden is completely defined. We are also are aware about the uncertainty around what constitutes clinical significance of PCa. For our analysis, we defined insignificant PCa as organ-confined Gleason 3 + 3 tumours, with no grade 4 or 5, the largest tumour having a volume ≤ 1.3 cm^3^ and a total tumour volume of ≤2.5 cm^3^ according to the updated ERSPC definition.^3^ This data was defined by analysis of pathology specimens of a large patient cohort with long-term follow-up and who were diagnosed with PCa by PSA screening. The definition is applicable to clinical practice and represents the extent of our current knowledge. Others argue that tumour volume is not important and that any organ confined Gleason 3 + 3 is insignificant. Whilst this is controversial, we also tested the accuracy of the NRS in predicting organ confined Gleason 3 + 3 of any volume and compared it with the accuracy of the pre-existing tools we identified. The AUC for our tool was 0.717 compared with 0.57 for the risk score by Parker et al.^8^, and 0.54 for the risk score by Carter et al.^5^ (data not shown).

## Conclusion

Our NRS shows better predictive power for insignificant PCa than any of the existing risk scores we examined in terms of sensitivity-, specificity and AUC. It allows clinicians to select sensitivity and specificity thresholds to allow development of an individualised treatment strategy based on patient characteristics, such as comorbidity, age and compliance. Nonetheless, the decision on whether a patient should undergo radical treatment or AS, lies between the clinician and the patient after thorough counselling. Our study also confirms the need for further investigation with confirmatory biopsy and/or MRI prior to embarking an AS programme.

An online version of our NRS can be found here:

https://www.evidencio.com/models/show/1391

## Electronic supplementary material

Supplementary Figure 1

Supplementary Table 1

Supplementary Figure 2

Supplementary Table 2

Supplementary Figure 3

## Data Availability

Patient data is stored in the database of the Prostatazentrum Nordwest, St. Antonius-Hospital, Klinik für Urologie, Kinderurologie und Urologische Onkologie, Gronau, Germany.
