# Correction: Development and validation of a novel risk score for the detection of insignificant prostate cancer in unscreened patient cohorts

**DOI:** 10.1038/s41416-019-0408-7

**Published:** 2019-02-27

**Authors:** Lorenzo Dutto, Amar Ahmad, Katerina Urbanova, Christian Wagner, Andreas Schuette, Mustafa Addali, John D. Kelly, Ashwin Sridhar, Senthil Nathan, Timothy P. Briggs, Joern H. Witt, Gregory L. Shaw

**Affiliations:** 1 Prostatazentrum Nordwest, St. Antonius-Hospital, Klinik für Urologie, Kinderurologie und Urologische Onkologie, Gronau, Germany; 2 Department of Urology, Queen Elisabeth University Hospital, Glasgow, UK; 3 Wolfson Institute of Preventive Medicine, Barts and The London School of Medicine, Queen Mary University of London, Centre for Cancer Prevention, London, UK; 4 Department of Urology, University College London Hospital, London, UK

**Correction to:** *British Journal of Cancer* (2018) **119**:1445–1450; 10.1038/s41416-018-0316-2; www.bjcancer.com; published online 27 November 2018

Since the publication of this paper, the authors noticed that Amar Ahmad was not credited as contributing equally to the paper. He should be considered as a joint first author with Lorenzo Dutto. In addition, the author Ashwin Sridhar was incorrectly listed as Ashwin Shridhar, and the author Gregory L. Shaw was incorrectly listed as Gregory Shaw. The correct names are listed above.

