# Supplementary material for: Development and validation of a novel risk score for the detection of insignificant prostate cancer in unscreened patient cohorts
Source: Br J Cancer. 2018 Nov 27;119(12):1445–50. doi: 10.1038/s41416-018-0316-2 (PMC6288120; doi:10.1038/s41416-018-0316-2)
Supplement: Supplementary file 1 — Supplementary Figure 1 [file 41416_2018_316_MOESM1_ESM.docx]

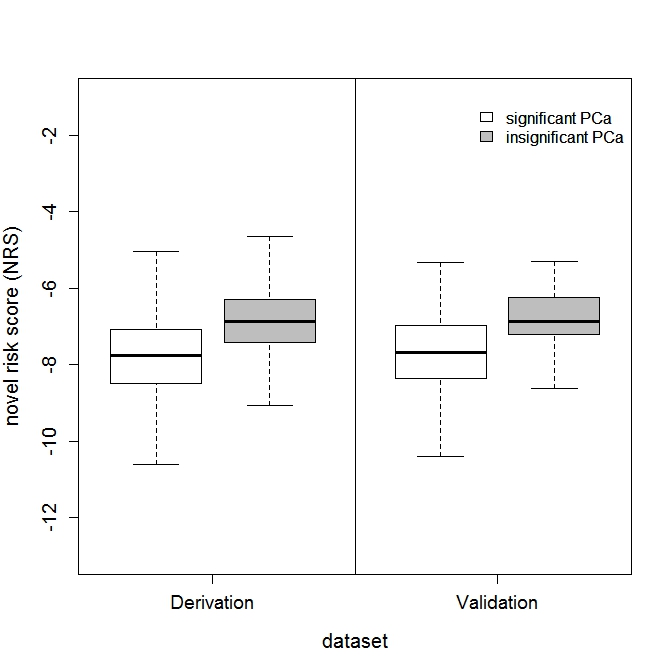


**Supplementary Figure 1:** distribution of the NRS in the derivation and validation datasets.

(Significant and insignificant PCa as per definition of the updated ERSPC prostate cancer risk criteria)
