# Supplementary material for: Development and validation of a novel risk score for the detection of insignificant prostate cancer in unscreened patient cohorts
Source: Br J Cancer. 2018 Nov 27;119(12):1445–50. doi: 10.1038/s41416-018-0316-2 (PMC6288120; doi:10.1038/s41416-018-0316-2)
Supplement: Supplementary file 2 — Supplementary Table 1 [file 41416_2018_316_MOESM2_ESM.docx]

| Population characteristics on pathology | | | | |
| --- | --- | --- | --- | --- |
|  | \| High risk PCa \| \| --- \| | \| Intermediate risk PCa \| \| --- \| | \| Low risk PCa \| \| --- \| | Total |
| n | 295 | 651 | 1853 | 2799 |
|  |  |  | Very low risk PCa |  |
|  |  |  | 969/1853 |  |
| Population clinical staging by digital rectal examination (DRE) | | | | |
|  | PCa risk group | Intermediate/high risk | Low risk | Total |
|  | Stage on DRE |  |  |  |
|  | cT1a | 3 | 5 | 8 |
|  | cT1b | 1 | 0 | 1 |
|  | cT1c | 1146 | 850 | 1996 |
|  | cT2a | 283 | 127 | 410 |
|  | cT2b | 204 | 39 | 243 |
|  | cT2c | 117 | 24 | 141 |
|  |  |  |  |  |
|  | Total | 1754 | 1045 | 2799 |

**Supplementary Table 1:** distribution of PCa risk groups and clinical stage on digital rectal examination
