# Supplementary material for: Development and validation of a novel risk score for the detection of insignificant prostate cancer in unscreened patient cohorts
Source: Br J Cancer. 2018 Nov 27;119(12):1445–50. doi: 10.1038/s41416-018-0316-2 (PMC6288120; doi:10.1038/s41416-018-0316-2)
Supplement: Supplementary file 3 — Supplementary Figure 2 [file 41416_2018_316_MOESM3_ESM.docx]

**
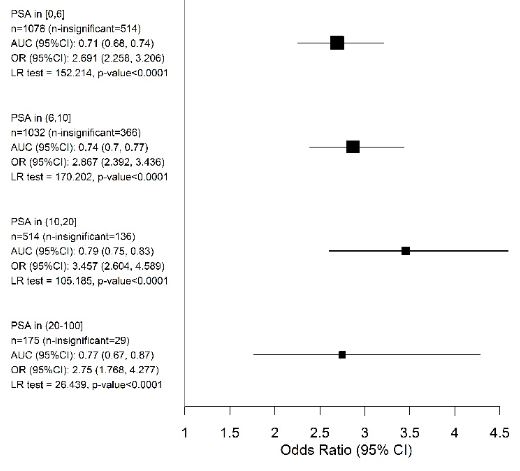
**

**Supplementary Figure 2:** Forest plot of the NRS stratified by PSA for detecting insignificant PCa (according to the updated ERSPC prostate cancer risk criteria). Subgroup analysis was performed in four PSA groups. Univariate logistic regression was performed in each PSA subgroup.

The NRS was statistically significant in each model with insignificant PCa=1 versus significant PCa=0. The odds ratio with 95% CI, likelihood ratio statistics and their corresponding p-value as well as the AUC were estimated from each fitted model. The size of each box is based on precision within that subgroup. The horizontal lines indicate the 95% CI for each odds ratio.
