# Supplementary material for: Development and validation of a novel risk score for the detection of insignificant prostate cancer in unscreened patient cohorts
Source: Br J Cancer. 2018 Nov 27;119(12):1445–50. doi: 10.1038/s41416-018-0316-2 (PMC6288120; doi:10.1038/s41416-018-0316-2)
Supplement: Supplementary file 4 — Supplementary Table 2 [file 41416_2018_316_MOESM4_ESM.docx]

| **Risk Score** | **Candidate clinical criteria to identify patients with insignificant PCa** |
| --- | --- |
| Schroder **^[3]^** | Baseline PSA≤10 & PSAD<0.2 & NPC≤2 & T-stage≤1c |
| Carter **^[5]^** | PSAD≤0.15 & NPC≤2 & PPC≤50 & T-stage≤1c |
| Parker **^[8]^** | baseline PSA≤15 & Gleason≤6 & T-stage≤2c & PPC≤50 |
| Carroll **^[13]^** | Baseline PSA≤10 & PNPC≤33 & PPC≤50 & T-stage≤1c |
| Eastham **^[6]^** | Baseline PSA≤10 & NPC≤3 & PPC≤50 & T-stage≤2a |
| Soloway **^[4]^** | Baseline PSA≤15 & NPC≤2 & PPC≤20 & T-stage≤1c |
| Very low * | Baseline PSA≤10 & NPC≤2 & T-stage≤2a & Gleason≤6 |
| Babaian **^[7]^** | Gleason≤6 & NPC≤2 |

**Supplementary Table 2:** summary of candidate clinical criteria by which to identify patients bearing insignificant PCa in existing risk assessment tools.

NPC: number of positive cores, PPC: percentage of positive cores

* Very low risk: D’Amico low risk with two or fewer biopsy cores containing cancer.
