# Supplementary material for: Development and validation of a novel risk score for the detection of insignificant prostate cancer in unscreened patient cohorts
Source: Br J Cancer. 2018 Nov 27;119(12):1445–50. doi: 10.1038/s41416-018-0316-2 (PMC6288120; doi:10.1038/s41416-018-0316-2)
Supplement: Supplementary file 5 — Supplementary Figure 3 [file 41416_2018_316_MOESM5_ESM.docx]

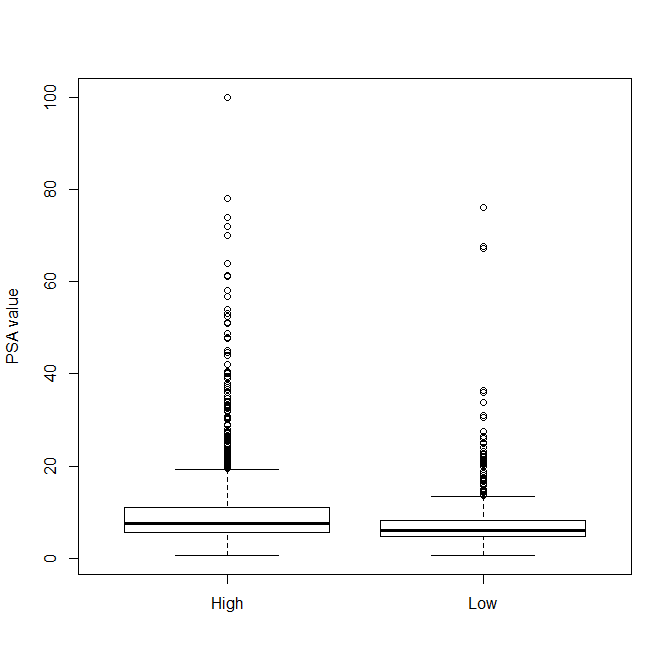


**Supplementary Figure 3:** distribution of PSA values amongst the study population.

High: high- and intermediate risk PCa

Low: low risk PCa

(risk groups are defined as per the definition of the updated ERSPC prostate cancer risk criteria)
